# Electrical Impedance Tomography Based on Grey Wolf Optimized Radial Basis Function Neural Network

**DOI:** 10.3390/mi13071120

**Published:** 2022-07-15

**Authors:** Guanghua Wang, Di Feng, Wenlai Tang

**Affiliations:** 1 Jiangsu Key Laboratory of 3D Printing Equipment and Manufacturing, School of Electrical and Automation Engineering, Nanjing Normal University, Nanjing 210023, China; 15152963380@163.com (G.W.); a15651730290@163.com (D.F.); 2 Jiangsu Key Laboratory for Design and Manufacture of Micro-Nano Biomedical Instruments, School of Mechanical Engineering, Southeast University, Nanjing 211189, China; 3 State Key Laboratory of Pharmaceutical Biotechnology, Department of Sports Medicine and Adult Reconstructive Surgery, Nanjing Drum Tower Hospital, The Affiliated Hospital of Nanjing University Medical School, Nanjing 210008, China; 4 Nanjing Institute of Intelligent High-End Equipment Industry Co., Ltd., Nanjing 210042, China

**Keywords:** electrical impedance tomography, grey wolf optimization algorithm, image reconstruction, radial basis function neural networks

## Abstract

Electrical impedance tomography (EIT) is a non-invasive, radiation-free imaging technique with a lot of promise in clinical monitoring. However, since EIT image reconstruction is a non-linear, pathological, and ill-posed issue, the quality of the reconstructed images needs constant improvement. To increase image reconstruction accuracy, a grey wolf optimized radial basis function neural network (GWO-RBFNN) is proposed in this paper. The grey wolf algorithm is used to optimize the weights in the radial base neural network, determine the mapping between the weights and the initial position of the grey wolf, and calculate the optimal position of the grey wolf to find the optimal solution for the weights, thus improving the image resolution of EIT imaging. COMSOL and MATLAB were used to numerically simulate the EIT system with 16 electrodes, producing 1700 simulation samples. The standard Landweber, RBFNN, and GWO-RBFNN approaches were used to train the sets separately. The obtained image correlation coefficient (ICC) of the test set after training with GWO-RBFNN is 0.9551. After adding 30, 40, and 50 dB of Gaussian white noise to the test set, the attained ICCs with GWO-RBFNN are 0.8966, 0.9197, and 0.9319, respectively. The findings reveal that the proposed GWO-RBFNN approach outperforms the existing methods when it comes to image reconstruction.

## 1. Introduction

Electrical impedance tomography (EIT) is a novel functional imaging approach that uses electrical information at field boundaries to reconstruct an image of the electrical conductivity distribution within an object [1]. Because of its benefits of radiation-free viewing, quick reaction, non-invasive and simple structure, the EIT technique has been a focus of study and is extensively employed in industrial processes [2] and medical monitoring [3,4].

According to Maxwell’s equations, the potential distribution measured from electrodes and the exciting current density determine the electrical conductivity distribution of the internal material. On the other hand, the image reconstruction of the EIT is a non-linear, pathological, and ill-posed issue [5].

The traditional image reconstruction methods can be divided into dynamic algorithms (isotropic inverse projection method [6], sensitive matrix method, and conjugate gradient method [7] and static algorithms (Gaussian Newton method [8], layer peeling method [9], static imaging algorithm for isotropic inverse projection, etc.). Dynamic algorithms are quicker to image and are often used for online reconstruction, but they need less precision from the data gathering apparatus and produce images of lower quality, as well. On the other hand, although the quality of reconstructed images generated using static methods has been improved, the repetitive search for incredibly small value points is computationally taxing, slowing down imaging and increasing noise sensitivity. Traditional image reconstruction techniques commonly utilize a linear equation to establish a mathematical model of the relationship between border voltage levels and conductivity distribution inside the object field [10]. The linearization procedure loses a lot of crucial information, resulting in substantial distortion of the rebuilt image.

Neural networks are distributed information storage structures that avoid the linearized analysis of sensitive matrix computing and image reconstruction by having huge parallelism, non-linearity, high self-adaptability, and strong self-learning capacity [11]. Many academics have committed themselves in recent years to solving the EIT image reconstruction challenge using various neural networks [12]. The BP neural network, convolutional neural network [13], and radial basis function are three typical effective and reliable neural network models.

A radial basis function neural network (RBFNN) is a high-performing feed-forward neural network. It has a remarkable global approximation capacity for nonlinear models, and can approximate any nonlinear function with arbitrary precision, which sets it apart from other neural networks. Additionally, due to its straightforward structure, rapid rate of learning convergence, and absence of sensitive matrix computation, the RBFNN satisfies the requirements of EIT image reconstruction [14]. The weights in RBFNN have a substantial influence on the network model’s overall performance and are directly connected to the predictability of the outcomes. When there is a lot of noise in the training data, the least squares method (LSM) will lead the neural network to fit an inaccurate surface. This will make the network less versatile. Furthermore, as the number of input samples increases, the disparity between the members of the generated weight matrix also increases. This will lead to an unstable solution, ultimately resulting in low-quality EIT reconstruction images.

A grey wolf optimized radial basis function neural network (GWO-RBFNN) is proposed in this paper to improve the network’s accuracy for image reconstruction of EIT in the presence of noise. The method adjusts the network center using the K-means algorithm and determines the network base width using the KNN (K-nearest neighbors) algorithm. Then, it uses the grey wolf optimization algorithm instead of the LSM to obtain more stable network weights, achieving the goal of improving the network model’s prediction accuracy. It was shown that the GWO-RBFNN approach proposed in this paper successfully improves the reconstruction quality of EIT images and boosts the artifact removal ability by comparing the reconstruction outcomes of other algorithms.

## 2. Theory

### 2.1. Mathematical Model

**Figure 1:**
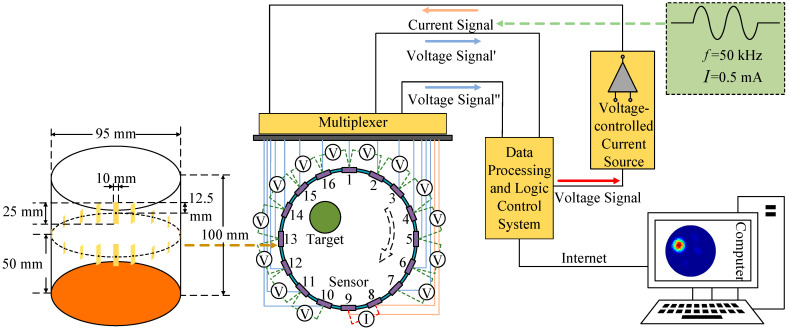
Diagram of EIT system.

The diagram of the EIT system is shown in Figure 1. There are 16 electrodes evenly distributed on the sensor. The electrode width in this work is 10 mm. The length of the electrode is 25 mm and the height of the electrode sensor is 100 mm. The geometric center of the electrode is located 50 mm from the electrode sensor.

When a safe AC excitation current signal is applied to the electrode sensor at the field boundary, the multiplexer can measure the voltage signal of the remaining electrode pairs at the field boundary, send the resulting analog signal to the data acquisition section, and then uses an image reconstruction algorithm and the collected voltage data to reconstruct the conductivity distribution inside the field [15].

The EIT measures the field domain, which satisfies Maxwell’s equations and electromagnetic field theory, and can be mathematically modeled as follows:

(1) ∇ · [σ(x,y)∇φ(x,y)]=0, (x,y)∈∂Ω

where Ω denotes the field, σ(x, y) indicates the internal conductivity distribution of the field, and φ(x, y) represents the distribution function of the field potential.

The EIT field boundary condition is set to:

(2) $$\frac{\sigma (x,y)\partial \phi (x,y)}{\partial n}=-j(x,y),\;(x,y)\in \partial \Omega$$

(3) ϕ(x,y)=U(x,y)

where ∂Ω denotes the field boundary, j(x, y) denotes the current density of the injected current on the boundary, *n* denotes the normal unit vector outside the field, and U(x, y) denotes the potential distribution at the field boundary.

The boundary excitation current *j* is chosen as a fixed value, and a constant current source is used as the excitation current in the experiment. The frequency and amplitude of the excitation current are constant, and then the image reconstruction becomes an investigation of the relationship between the conductivity distribution *σ* and the potential distribution ϕ in the field.

### 2.2. Building an RBFNN for EIT

**Figure 2:**
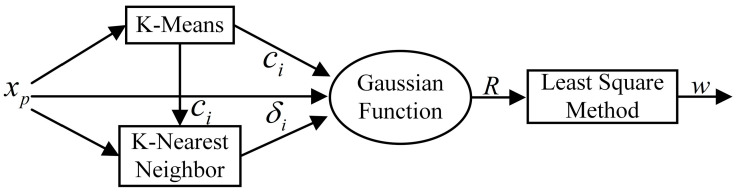
The classical RBFNN algorithm flow. The K-means algorithm is used to adjust the network center, the KNN algorithm to determine the network base width, and the LSM algorithm to calculate the connection weights to finally obtain the predicted conductivity of the EIT reconstructed image.

The fundamental construction of an RBFNN is shown in Figure 2, which is a kind of forward neural network. It is made up of three layers: the input layer, the concealed layer, and the output layer. The signal is sent from the input layer to the hidden layer, which completes the non-linear transformation from the input layer to the hidden layer space by using the radial basis function as the activation function. The RBFNN can approximate any non-linear function, which not only speeds up convergence and eliminates the issue of local minima, but also fits the EIT image reconstruction requirements.

In the network operation structure, the input layer is ${X=[x}_{1},\cdots {,x}_{p}{]}^{T}$. When a Gaussian function is used for the basis function in the radial basis neural network, the predicted conductivity ${Y=[y}_{1},\cdots {,y}_{n}{]}^{T}$ can be expressed as:

(4) $$y_{n}=\sum_{i=1}^{n}w_{n}\exp\left(-\frac{1}{2b_{i}^{2}}{\left\|x_{p}-c_{i}\right\|}^{2}\right)$$

where $\left\|x_{p}-c_{i}\right\|$ is the Euclidean parametrization, ${C=[c}_{1},\cdots {,c}_{i}{]}^{T}$ is the center of the Gaussian function, ${B=[b}_{1},\cdots {,b}_{i}{]}^{T}$ is the base width vector, and ${W=[w}_{1},\cdots {,w}_{n}{]}^{T}$ is a vector of connection weights.

The center vector of the function, the base width vector, and the vector of weights from the hidden layer to the output layer are the unknown parameters that the network must learn. The following are the stages in its learning algorithm:

1. Finding the center of the basis function based on the K-means method.

The center can be adjusted by the following formula:

(5) $$c_{i}^{t+1}={\{}_{c_{i}^{t},\;j\neq j(X_{p}^{t})}^{c_{i}^{t}+\eta (X_{p}^{t}-c_{i}^{t}),\;j=j(X_{p}^{t})}$$

where $X_{p}^{t}$ is the training sample vector of the input *p*, $c_{i}^{t}$ is the *i* center of RBF at the *t* iteration, *j* is the cluster center, η is the iteration step, and 0 < η < 1. After learning all the training samples, and when the cluster center change satisfies the iteration condition, the iteration stops.

2. Solving for variance $b_{i}$.

The basis function of this neural network is a Gaussian function, and the variance can be solved as follows:

(6) $$b_{i}=\frac{c_{\max}}{\sqrt{2h}},\;i=1,2,\ldots ,h$$

where $c_{\max}$ is the maximum distance between the selected centers.

3. Calculating the weights between the implied and output layers.

The weights of neuron connections between the implicit layer and the output layer are directly calculated by the LSM with the following equation:

(7) $$w_{n}=\exp\left(\frac{h}{c_{\max}^{2}}{\left\|x_{p}-c_{i}\right\|}^{2}\right),\;i=1,2,\ldots ,p$$

Through the above learning steps, the learning algorithm of RBFNN is constructed.

## 2.3. Grey Wolf Algorithm for Optimizing RBFNN Models

**Figure 3:**
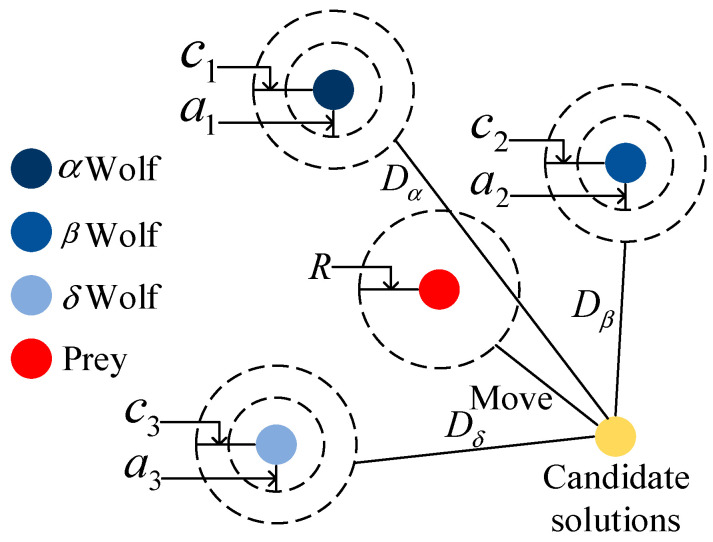
The optimization process of the grey wolf optimization algorithm. Taking α as the most suitable solution, the three wolves are guided by α, β, and δ during the hunting process, and wolf ω follows these three wolves.

To improve the accuracy of RBFNN reconstructed images, we propose to use the improved grey wolf optimization algorithm to obtain stable network weights. Firstly, it is necessary to construct a grey wolf social hierarchy model when designing the GWO algorithm. Secondly, we should calculate the fitness of each individual in the population. Lastly, we should mark the three grey wolves with the best fitness in the pack as α, β, and δ in turn, while the remaining grey wolves are marked as ω. The hunting behavior is shown in Figure 3.

The behavior of the grey wolf for hunting its prey is defined as shown in Equations (8) and (9):

(8) $$D=\left|C\;\cdot \;X_{p}\left(t\right)-X\left(t\right)\right|$$

(9) $$X\left(t+1\right)=X_{p}(t)-A\;\cdot \;D\;$$

where Equation (8) represents the distance between an individual and its prey, and Equation (9) is the position update formula for the grey wolf, where *t* is the number of generations of the current iteration, *A* and *C* are the coefficient vectors, and *X* and $X_{p}$ are the position vector of the prey and the position vector of the grey wolf, respectively. *A* and *C* are calculated as follows:

(10) $$A=2a\;\cdot \;r_{1}-a$$

(11) $$C=2\;\cdot \;r_{2}$$

where *a* is a convergence factor that decreases linearly from 2 to 0 with the number of iterations, and the norms of $r_{1}$ and $r_{2}$ fall into a random number between [0, 1]. The *C* vector provides random weights for the prey. In order to simulate approaching prey, the value of *a* is gradually reduced. During the iterations, as the value of *a* decreases linearly from 2 to 0, and its corresponding value of *A* also varies within the interval $\left[-a,\;a\right]$.

**Figure 4:**
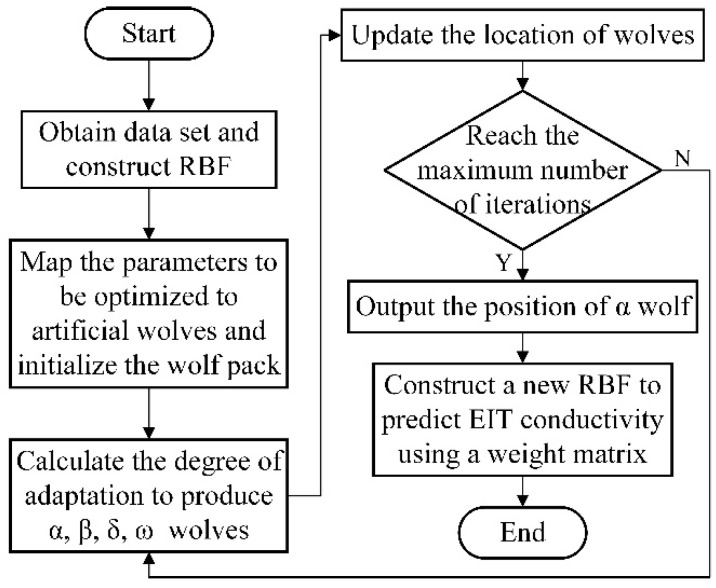
Flowchart of the EIT algorithm based on the proposed GWO-RBFNN. Using the optimized algorithm to calculate the internal conductivity of the EIT results in a reconstructed image.

The connection weights of RBFNN are crucial parameters that directly affect the reliability of the predicted EIT reconstruction results. The LSM algorithm subjectively regards the mapping from the implicit layer to the output layer as a linear mapping and needs to calculate the inverse matrix, ignoring the circumstance that the inverse matrix does not exist. To solve this problem, we optimize the connection weights *W* to improve the prediction accuracy of RBFNN through the GWO algorithm, instead of calculating the inverse matrix. The specific optimization process of the proposed algorithm is shown in Figure 4, and described as follows:

(1) The EIT using 16 electrodes was modeled and simulated. The dataset was acquired using COMSOL in combination with MATLAB simulation and then separated into a test and training set based on this model.

(2) Initializing the grey wolf algorithm. Firstly, the positions of the individual artificial grey wolves are generated randomly in the definition domain. Secondly, a mapping between the grey wolf position dimensions and the connection weights *W* is established. Lastly, the weight matrix *W* from the hidden layer to the output layer of the neural network is mapped into the position vector of the artificial grey wolves to construct the RBFNN model.

(3) Calculating the fitness. The RMSE (root mean square error) of the output of the neural network, as described in Equation (12). It is used as the fitness function of the grey wolf algorithm. The fitness function is a measure of the merit of the position of the individual grey wolf. The smaller the value of the fitness function *S*, the better the position, which is defined as follows:

(12) $$S=\sqrt{\frac{1}{n}\sum_{i=1}^{N}{\left(D_{i}-Y_{i}\right)}^{2}}$$

where $Y_{i}$ is the training output, $D_{i}$ is the expected value, and *N* is the capacity of the entire training sample.

(4) Updating the location of the grey wolf. The optimal wolf position is calculated by the grey wolf algorithm and remapped to the connection weights of the RBFNN hidden layer to the output layer.

(5) The end condition is satisfied and the iteration is stopped. The optimized weights are obtained, and the optimal solution is applied to the RBFNN. Then, the trained RBFNN model is obtained by inputting the optimized weights into the training set, and the reconstructed image of the EIT is predicted using the test set.

## 3. Results

### 3.1. Acquisition of Datasets

The quality of the dataset has a substantial influence on the network model’s generalization capability, and neural network learning needs a high number of samples to train the network model. Simulation datasets were created using combined COMSOL and MATLAB simulations. They will be used to solve the issue, which is a large number of samples with actual conductivity distributions and accompanying boundary voltage measurements not being accessible in real systems.

**Figure 5:**
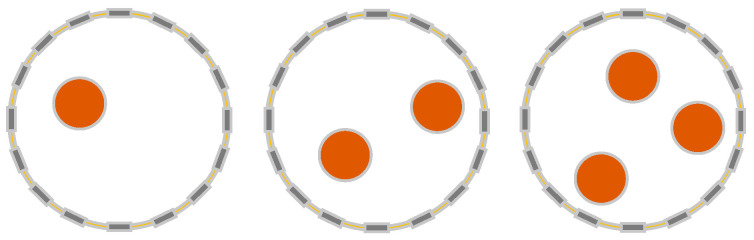
The different EIT configurations for the simulations.

A 16-electrode configuration is chosen in this work because a 16-electrode EIT system has been widely used with a satisfactory resolution. Then, using a 16-electrode EIT system with adjacent current excitation and adjacent voltage measurement modes, simulations were run to reconstruct the field’s internal conductivity distributions. When choosing the target shape, we need to consider the convenience between the target and the reconstructed image to highlight the effect of the algorithm optimization. Compared to other shapes of targets, circles have very good image reconstruction results. Therefore, the classical circular target object is chosen. Circular targets with a diameter of 10 mm were randomly formed in a circular physical field with a diameter of 95 mm, as shown in Figure 5. When the difference between the conductivity of the background solution and the conductivity of the target is greater, the more sensitive the change in boundary voltage is. Therefore, the better the image reconstruction will be. Thus, in order to obtain good image reconstruction, the conductivity of the background solution was set to $5{.5\;\times \;10}^{-4}$ S/m and the conductivity of the circular target was chosen as $5{.5\;\times \;10}^{-8}$ S/m. To obtain the internal conductivity distributions, the 0.5 mA excitation current was applied, and the measurement frequency was set to 50 kHz. Since the targets in this paper are basically near the boundary, the adjacency excitation method was chosen so high sensitivity to changes in conductivity near the boundary can be obtained.

Single, double, and triple circular targets were investigated. To obtain the conductivity distributions and accompanying boundary voltage values, each group underwent 1700 numerical simulations with varied target locations. The remaining 200 samples were utilized for testing, while 1500 samples were used for training. This ensured that the training and testing sets did not overlap.

### 3.2. Simulation Results

**Figure 6:**
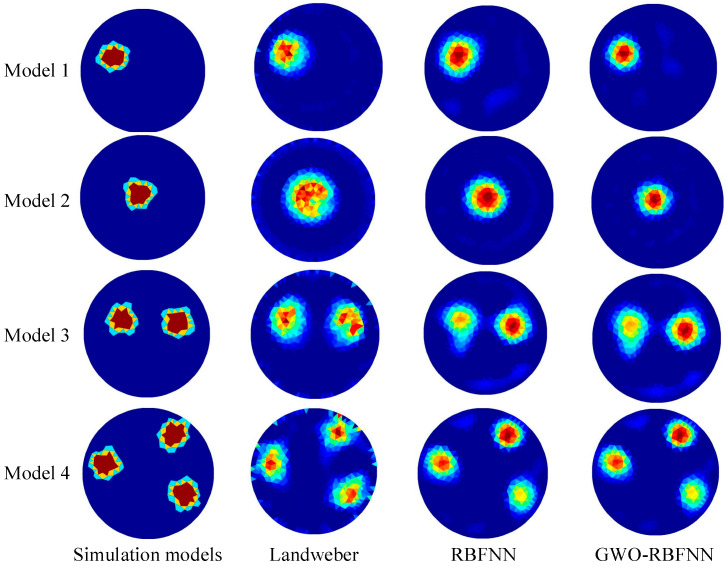
Typical models from the noise-free test set and their reconstructed images with different algorithms.

The improved model was then utilized to predict the reconstructed images of the test sets after training with 1500 samples. Typical models from the noise-free test set and their reconstructed images with different algorithms are shown in Figure 6. Two commonly-used algorithms, Landweber and RBFNN, were also employed for comparison to demonstrate the prediction accuracy of the proposed GWO-RBFNN. The findings reveal that all of the algorithms can reassemble images of targets in various configurations. At the same time, the proposed GWO-RBFNN outperforms the other two algorithms in terms of prediction precision, especially when a single circular target is positioned in the center of the background solution.

The RMSE and the ICC were selected as the rating criteria to quantitatively assess the image reconstruction quality of various algorithms. The RMSE gives a good indication of the accuracy of the observations. The smaller the value of RMSE, the better the reconstruction of the conductivity value. On the other hand, the closer the correlation coefficient is to 1 or −1, the stronger the correlation. The closer the correlation coefficient is to 0, the weaker the correlation. They are described by the following formulae:

(13) $$RMSE=\sqrt{\frac{1}{n}\sum_{i=1}^{N}{\left(Y_{i}^{*}-Y_{i}\right)}^{2}}$$

(14) $$ICC=\frac{\sum_{i=1}^{n}\left(Y_{i}^{*}-\bar{Y^{*}}\right)\left(Y_{i}-\bar{Y}\right)}{\sqrt{\sum_{i=1}^{n}{\left(Y_{i}^{*}-\bar{Y^{*}}\right)}^{2}\sum_{i=1}^{n}{\left(Y_{i}-\bar{Y}\right)}^{2}}}$$

where $Y_{i}^{*}$ and $\bar{Y^{*}}$ are the estimated conductivity and its average value, respectively; $Y_{i}$ and $\bar{Y}$ are the original conductivity and its average value, respectively; and *n* is the number of elements in the finite element model. The estimated RMSE and ICC averages of the reconstructed images based on Equations (13) and (14) for all test sets with various approaches are shown in Table 1. The proposed GWO-RBFNN approach clearly outperforms the Landweber and RBFNN algorithms in terms of image reconstruction quality, with the lowest RMSE value of 0.0848 and the greatest ICC value of 0.9519. The values of RMES decreased by 46.3% and 20.2%, respectively. The values of ICC improved by 14.4% and 9%, respectively.

### 3.3. Robustness of the GWO-RBFNN

**Figure 7:**
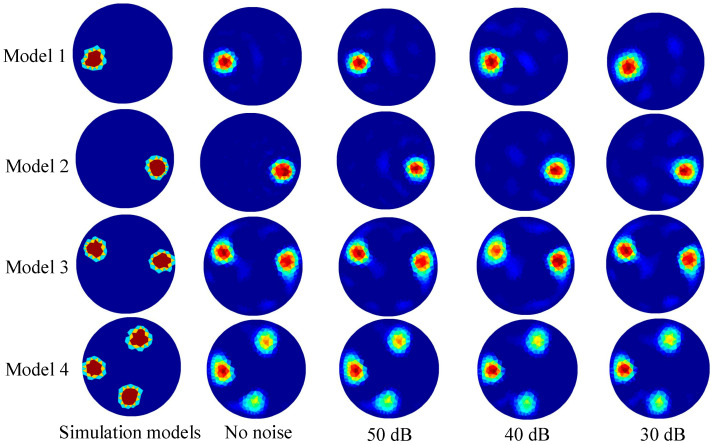
The reconstructed images of typical models with different noise levels using the proposed GWO-RBFNN.

To test the robustness of the proposed method against noise, Gaussian white noises of 30 dB, 40 dB, and 50 dB were added to the test sets. Exemplary models with various Gaussian white noises created using the proposed GWO-RBFNN are shown in Figure 7. The findings reveal that all of the models have high-quality image reconstructions.

The average values of RMSE and ICC derived by the three alternative approaches, with varied noise levels in the test sets, are shown in Table 2 and Table 3. The simulation results with noise show that when different levels of noise are applied to the test set, the average RMSE increases while the average ICC decreases. Under Gaussian white noises of 30 dB, 40 dB, and 50 dB, the RMSEs of the proposed approach in this paper are raised from 0.0848 to 0.1139, 0.0962, and 0.0915, respectively. They increased by 34.3%, 13.4%, and 7.9% respectively. The ICCs of the proposed method decreased from 0.9551 to 0.8966, 0.9197, and 0.9319, respectively. They decreased by 6.1%, 3.7%, and 2.4%, respectively. However, it can be seen that all the RMSEs with the proposed GWO-RBFNN are lower than the other two algorithms, and all the ICCs with the proposed GWO-RBFNN are higher than the other two algorithms. The results show that the algorithm proposed in this paper exhibits better robustness than the frequently used Landweber and RBFNN algorithms.

From Figure 6 and Figure 7, it can be seen that the GWO-RBFNN approach developed in this work not only has some noise immunity, but it also has some generalization capacity to adapt to the scenario of multi-target detection. According to the image reconstruction results of the multi-target test sets, when the GWO-RBFNN approach is used for multi-target imaging, the number of target objects is clearly recognized, and the position and size of the detected targets can be correctly displayed. The related RMSE and ICC averages still provide more acceptable results without considerable deterioration, indicating that the approach proposed in this paper is capable of satisfactory generalization.

## 4. Conclusions

A GWO-RBFNN approach is proposed in this paper to increase the accuracy of EIT image reconstruction. In order to improve the prediction accuracy of the network model, we first use the K-means method to adjust the network center. Then, we use the KNN algorithm to determine the network base width, and finally, we use the grey wolf algorithm to optimize the connection weight. A joint simulation using COMSOL and MATLAB was constructed to obtain 1700 EIT simulation samples for training and testing the performance of the proposed method. The image reconstruction results with noisy test sets demonstrate the robustness and generalization of the proposed GWO-RBFNN method. The GWO-RBFNN approach provides superior image reconstruction outcomes and artifact removal capacity compared to the Landweber and RBFNN methods, according to test findings from the 16-electrode EIT system.

## Figures and Tables

**Table 1:** Averages of RMSE and ICC in the noiseless test set.

Average	RMES	ICC
Landweber	0.1579	0.8347
RBFNN	0.1062	0.8756
GWO-RBFNN	0.0848	0.9551

**Table 2:** Average RMSE with noise test set.

Averages	No Noise	50 dB	40 dB	30 dB
Landweber	0.1579	0.1778	0.1974	0.2254
RBFNN	0.1062	0.1029	0.1045	0.1151
GWO-RBFNN	0.0848	0.0915	0.0962	0.1139

**Table 3:** Average ICC with noise test set.

Averages	No Noise	50 dB	40 dB	30 dB
Landweber	0.8347	0.8347	0.7383	0.5252
RBFNN	0.8754	0.8656	0.8325	0.8284
GWO-RBFNN	0.9551	0.9319	0.9197	0.8966

## Data Availability

Not applicable.
